# Veno-arterial extracorporeal membrane oxygenation supported transcatheter aortic valve implantation in a high-risk COVID-19 patient: a comprehensive case report

**DOI:** 10.1093/ehjcr/ytae457

**Published:** 2024-09-26

**Authors:** Giampiero Vizzari, Tommaso De Ferrari, Francesco Costa, Nastasia Mancini, Marco Franzino, Fabrizio Ceresa, Francesco Patanè, Antonio Micari

**Affiliations:** Interventional Cardiology Unit, ‘G. Martino’ University Hospital, Via C. Valeria, 1, 98125 Messina, Italy; Interventional Cardiology Unit, ‘G. Martino’ University Hospital, Via C. Valeria, 1, 98125 Messina, Italy; Interventional Cardiology Unit, ‘G. Martino’ University Hospital, Via C. Valeria, 1, 98125 Messina, Italy; Interventional Cardiology Unit, ‘G. Martino’ University Hospital, Via C. Valeria, 1, 98125 Messina, Italy; Interventional Cardiology Unit, ‘G. Martino’ University Hospital, Via C. Valeria, 1, 98125 Messina, Italy; Cardio-Thoraco-Vascular Department, Division of Cardiac Surgery, Papardo Hospital, Messina, Italy; Cardio-Thoraco-Vascular Department, Division of Cardiac Surgery, Papardo Hospital, Messina, Italy; Interventional Cardiology Unit, ‘G. Martino’ University Hospital, Via C. Valeria, 1, 98125 Messina, Italy

**Keywords:** Transcatheter aortic valve implantation (TAVI), High-risk TAVI, Extracorporeal membrane oxygenation (ECMO), Mechanical circulatory support (MCS), COVID-19 (or SARS-CoV2), Case report

## Abstract

**Background:**

The sudden onset of heart failure in high-risk transcatheter aortic valve implantation (TAVI) candidates poses significant challenges, necessitating meticulous planning and consideration of mechanical circulatory support options. Nevertheless, existing data on the efficacy and safety of mechanical circulatory support in this context are limited, along with criteria for patient selection.

**Case summary:**

An 87-year-old patient, with severe low-flow low-gradient aortic stenosis, presented with acute heart failure and concurrent COVID-19 pneumonia. Despite initial conservative management, her clinical condition deteriorated, requiring inotropic support. The decision was made to perform a rescue TAVI procedure with veno-arterial extracorporeal membrane oxygenation (ECMO) support. The patient underwent successful TAVI while managing complications, including cardiac arrest, with haemodynamic support from veno-arterial ECMO. Post-procedure, the patient showed improved cardiac function and was discharged in stable condition.

**Discussion:**

This case underscores the significance of strategic patient selection, proactive haemodynamic management, and the judicious use of veno-arterial ECMO in high-risk TAVI, particularly in complex scenarios involving acute heart failure and respiratory insufficiency, exacerbated by COVID-19. It highlights the challenges and critical decision points in TAVI planning, emphasizing the need for further research and standardized guidelines to refine indications for prophylactic mechanical circulatory support in TAVI procedures.

**Learning points**

- Utilizing veno-arterial extracorporeal membrane oxygenation (VA-ECMO) as prophylactic support in high-risk transcatheter aortic valve implantation (TAVI), especially in patients with combined left ventricular and respiratory failure, can be a strategic decision, substantiated by successful outcomes in this case.
- In COVID-19 patients with acute decompensated heart failure and severe aortic stenosis, the decision to perform TAVI with VA-ECMO support requires a delicate balance between optimal timing and patient status.

## Introduction

The sudden occurrence of heart failure is a serious risk for patients undergoing transcatheter aortic valve implantation (TAVI).^1^ To address such challenges, percutaneous mechanical circulatory support (MCS) devices, notably veno-arterial extracorporeal membrane oxygenation (VA-ECMO) and Impella (Abiomed, Danvers, MA), have been employed as backup measures in high-risk TAVI procedures.^2–7^ Among the two, VA-ECMO is the most suitable device for prophylactic use, as it leaves the aortic root free for the TAVI procedure, and provides respiratory support in cases of concurrent respiratory failure.^8–10^ Nevertheless, existing data on the efficacy and safety of MCS in this context are limited, and current guidelines defining the circumstances and patient profiles warranting prophylactic MCS are lacking.^11^

## Summary figure

Panel depicting decision-making process for the use of prophylactic ECMO support in high-risk TAVI patients.

**Figure:**
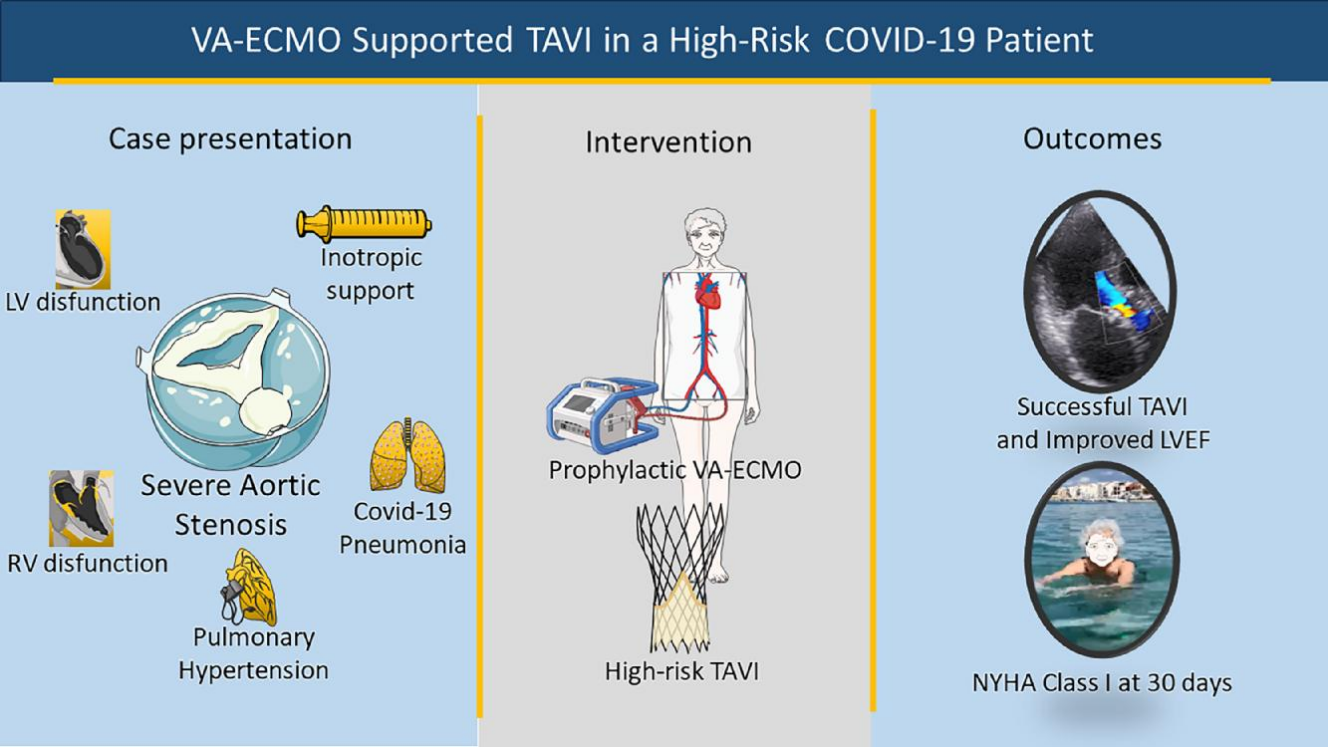


## Case report

An 87-year-old woman, with a history of dyslipidaemia and recent onset atrial fibrillation on oral anticoagulation, presented to our emergency department with dyspnoea and chest pain. Due to her positivity for COVID-19 and respiratory distress, she was admitted to the COVID-19 unit. The computed tomography (CT) scan of the chest displayed several areas of ground glass opacity and lung consolidations, together with bilateral pleural effusion (see Supplementary material online, *Video S1*). Simultaneously, it revealed severe calcification of the aortic valve and aortic root.

Subsequent echocardiogram evaluation showed a severely reduced left ventricular ejection fraction (LVEF 20–25%) with diffuse hypokinesia, right ventricular dysfunction (tricuspid annular plane excursion 12 mm), and severe low-flow low-gradient severe aortic stenosis (aortic valve area index 0.2 cm^2^) with associated moderate regurgitation, moderate mitral regurgitation, and moderate to severe tricuspid regurgitation with pulmonary hypertension (*Video 1*). It represented the first occurrence of heart failure, since her previous echocardiogram from 2 years earlier had shown a preserved LVEF (50%) and moderate aortic stenosis, which was lost at follow-up.

During the hospitalization in the COVID unit, the patient required non-invasive ventilation and inotropic support. After 37 days, with a negative COVID-19 test but still active pneumonia, she was transferred to our coronary care unit. She underwent coronary angiography, which showed no stenoses in the epicardial coronary arteries (see Supplementary material online, *Video S2*).

In the following days, the patient’s haemodynamic conditions deteriorated, with the occurrence of pulmonary oedema and signs of low cardiac output requiring inotropic support with dobutamine. Her haematochemical parameters demonstrated multifactorial anaemia (Hb 9 g/dL), normal platelet count (150 × 10^9^ L), chronic kidney disease (eGFR 40 mL/min/1.73 m^2^), elevated NT-proBNP (5000 pg/mL), and a normal coagulation profile.

**Figure 1:**
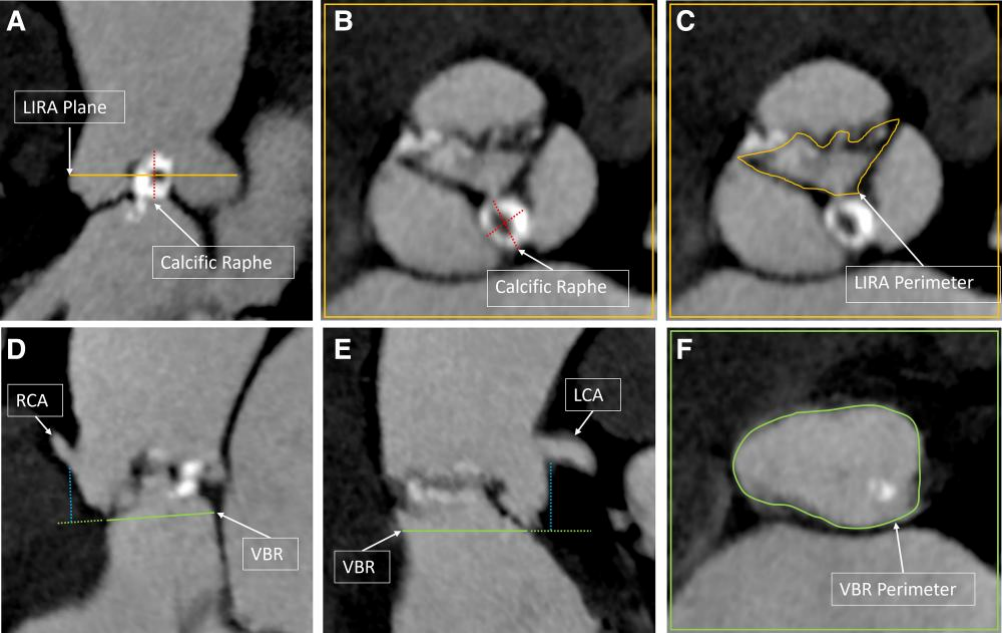
Pre-transcatheter aortic valve replacement aortic computed tomography scan. (*A* and *B*) Longitudinal and axial view at the raphe level (Level of Implantation at the RAphe plane, orange horizontal line, *B*) showing a bicuspid aorta root anatomy (Sievers Type 1) with a 9 × 7 mm calcific raphe (red dotted lines, *A* and *B*) between non-coronary cusp and left coronary cusp leaflets. (*C*) Prediction of the perimeter occupied by a transcatheter heart valve at the Level of Implantation at the RAphe according to the Level of Implantation at the RAphe method: Level of Implantation at the RAphe perimeter 82 mm (orange closed curve, *C*). (*D* and *E*) Longitudinal views at the virtual basal ring showing high coronary ostia (left 15 mm, right 14 mm) and wide Valsalva sinus. (*F*) Virtual basal ring measurements (green oval cuve, *F*: perimeter 92 mm, area 620 mm^2^), compatible with the largest available sizes of transcatheter heart valves. RCA, right coronary artery; LCA, left coronary artery; LIRA, Level of Implantation at the RAphe; VBR, virtual basal ring.

**Figure 2:**
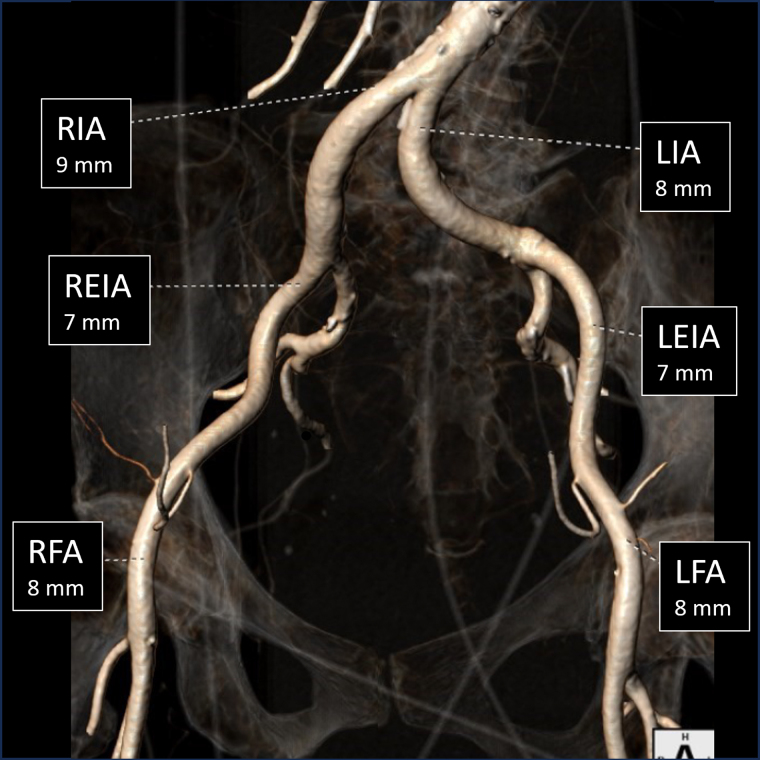
Computed tomography angiography of peripheral accesses. 3D reconstruction of the iliac and femoral arteries displaying access practicability. RIA, right iliac artery; REIA, right external iliac artery; RFA, right femoral artery; LIA, left iliac artery; LEIA, left external iliac artery; LFA, left femoral artery.

Given the rapid deterioration of her clinical condition and the inadequate response to medical therapy, we opted to plan a rescue TAVI procedure with VA-ECMO support (CardioHelp System, MAQUET, Rastatt, Germany). Computed tomography angiography of the aortic valve, performed with low-dose contrast injection during amine infusion, revealed a severely calcific aortic valve (calcium score 2786 AU), functionally bicuspid due to massive calcific commissural fusion between the non-coronary and left coronary cusp leaflets [Sievers Type 1, with a 9 × 7 mm calcific nodule between non-coronary cusp (NCC) and left coronary cusp (LCC) leaflets] (*Figure 1A* and *B*). Sinuses of Valsalva were wide, the coronary ostia were high (*Figure 1E* and *F*), and the virtual basal ring (VBR, area 620 mm^2^, perimeter 92 mm) was compatible with the largest available sizes of transcatheter heart valves (THVs) (*Figure 1F*). An urgent TAVI procedure was performed under deep sedation and low-dose inotropic support. Pre-procedure, invasive pressure measured 95/65 mmHg. Veno-arterial extracorporeal membrane oxygenation cannulation was performed through a left surgical cut-down veno-arterial femoral access (19 and 15 Fr for venous and arterial cannulae, *Figure 2*). Intravenous unfractionated heparin was used for anticoagulation during ECMO support. A 34 mm self-expandable CoreValve Evolut R prosthesis (Medtronic Inc., Minneapolis, MN, USA) was implanted through percutaneous right femoral access. Immediately after the THV deployment, the patient experienced a gradual bradycardia ending with a transient asystole, leading to a brief cardiac arrest. This was attributed to the under-expansion of the valve, caused by significant calcification and resulting in moderate-to-severe aortic regurgitation (*Video 2, left panel*). Fortunately, the haemodynamic support provided by the ECMO system, with a mean flow of 3.0–3.5 L/min at this stage, avoided complications from the cardiac arrest for the patient. Subsequently, we finalized the procedure by post-dilating the prosthesis using a 24 mm valvuloplasty balloon, achieving a good expansion of the prosthesis and a trivial residual paravalvular leak (*Video 2, central and right panel*). A second cardiac arrest occurred after the post-dilation of the prosthesis, presenting with a pulseless electrical activity rhythm, likely due to myocardial stunning during balloon inflation and rapid ventricular pacing. Invasive arterial pressure monitoring indicated no pulsatility despite ongoing electrical activity. Consequently, VA-ECMO flow was increased to 3–4 L/min, allowing normal ventricular mechanical activity to restore. The patient’s haemodynamic status stabilized.

Percutaneous vascular access for transcatheter aortic valve replacement was closed with double pre-positioned Perclose ProGlide Closure System (Abbott Vascular). Extracorporeal membrane oxygenation was removed the same day, and inotropic therapy was rapidly discontinued. Neither vascular complications were reported nor was a pacemaker implantation needed. The neurological status remained stable during the procedure, with no cerebral complications. Pre-discharge echocardiography confirmed the absence of aortic regurgitation with a normal trans-prosthetic gradient (mean gradient 10 mmHg) and revealed an improvement in the ejection fraction (LVEF 35%) with mild mitral and tricuspid regurgitation (*Video 3*). The patient was discharged in good clinical status [New York Heart Association (NYHA) II], on oral anticoagulation and complete heart failure therapy (beta-blockers, angiotensin-converting enzyme inhibitors, mineralocorticoid receptor antagonists, and SGLT2 inhibitor). One month after discharge, at her first follow-up examination, the patient was asymptomatic, in excellent clinical conditions (NYHA I), and echocardiography revealed a further slight improvement in left ventricular systolic function (LVEF 40%). The 1-year follow-up visit confirmed the proper performance of the aortic THV with a mild paravalvular leak and a mildly reduced LVEF.

## Discussion

The evolution of TAVI, coupled with increased experience and success rates, has expanded its applicability to very high-risk patients. Ensuring optimal outcomes in high-risk procedures requires meticulous preparation, incorporating maximal prophylactic support. However, the use of percutaneous MCS devices can be challenging due to their expense, limited availability, and potential complications.

Excluding the intra-aortic balloon pump (IABP), VA-ECMO emerges as the predominant MCS device during high-risk TAVI, offering simultaneous pulmonary support without interfering with the TAVI procedure.^6–8,12^ The MUST Registry, in accordance with the literature review conducted by Raffa *et al*.,^6^ confirmed lower peri-procedural mortality in high-risk patients with prophylactic percutaneous MCS (pMCS) use, primarily ECMO, compared to its use in emergency settings.^7^ Both studies identify lower LVEF, significant right ventricular dysfunction, and severe pulmonary hypertension as characteristics of high-risk TAVI patients who received prophylactic pMCS compared to those who underwent emergent pMCS. The patient we presented exhibited all the characteristics indicative of high-risk TAVI. In addition, she required inotropic support (with the Society for Cardiovascular Angiography and Interventions SHOCK Stage Classification moving between A to C during hospitalization) in conjunction with respiratory failure due to COVID-19.^13^ Physiologically, severe left ventricular failure and significant aortic insufficiency are haemodynamically unfavourable for VA-ECMO due to increased left ventricular afterload, left ventricular dysfunction, elevated left ventricular end-diastolic pressure, and pulmonary congestion. To mitigate these effects, we maintained VA-ECMO low-flow settings (around 2 L/min) before valve implantation and subsequent cardiac arrest.

In the decision-making process leading to TAVI, we considered the concomitant moderate aortic regurgitation, which contraindicated balloon aortic valvuloplasty. Simultaneously, a crucial aspect in favour of proceeding with TAVI was evaluating the patient’s optimal functional status before hospitalization. Recognizing the central role of aortic stenosis in the pathogenesis of clinical deterioration, deemed potentially reversible, guided our ultimate decision.

In the planning phase of the TAVI procedure, we found that the dimensions of the VBR were suitable for the Evolut 34 mm, as well as the balloon-expandable SAPIEN 3 29 mm (Edwards Lifesciences Inc., Irvine, CA) and Myval 29 mm (Meril Life Sciences Pvt Ltd, India). A self-expandable device was chosen over a balloon-expandable device due to the severe calcification of the valve and the associated risks of displacement of calcific nodules between the NCC and LCC leaflets, as well as potential aortic root injury. The decision to avoid pre-dilatation of the severely calcified bicuspid aortic valve was made to prevent worsening of the already moderate aortic insufficiency. At the raphe level (Level of Implantation at the RAphe plane), we observed that the perimeter and area were smaller than the VBR measurements, indicating the characteristic ‘volcano’ shape of bicuspid aortic valve disease in the aortic root^14^ (*Figure 1C*), not necessitating downsizing the THV. Undoubtedly, the prophylactic ECMO support proved essential in managing life-threatening complications throughout the critical phases of the procedure, including THV deployment and post-dilation. The initial cardiac arrest during valve implantation, triggered by acute aortic insufficiency due to THV under-expansion on the calcified raphe, was effectively addressed. The subsequent cardiac arrest, characterized by a pulseless electrical activity rhythm, was probably linked to myocardial stunning following high-frequency pacing during balloon inflation. The swift improvement in haemodynamic, clinical, and functional parameters, coupled with the enhanced left ventricular systolic function, emphasized the central role of aortic valve disease in precipitating acute heart failure.

## Conclusion

This case emphasizes the importance of strategic patient selection, proactive haemodynamic management, and judicious use of VA-ECMO, in high-risk TAVI procedures. The decision-making process should not only account for anatomical considerations but also address physiological conditions to ensure optimal outcomes in challenging cases. Further research and standardized guidelines are essential to refine indications for prophylactic pMCS in TAVI procedures.

## Supplementary Material

ytae457_Supplementary_Data

## Data Availability

The data underlying this article are available in the article and in its online Supplementary material.

## Lead author biography

Dr. Giampiero Vizzari is PhD and interventional cardiologist at the ‘G. Martino’ University Hospital of Messina, Italy. He trained as research fellow at the Aurora Healthcare Medical Center of Milwaukee, USA, and interventional cardiology fellow directed by Prof. F. Versaci. He worked as interventional cardiologist at ‘Giovanni Paolo II’ Hospital of Ragusa and ‘Umberto I’ Hospital of Siracusa. In 2022, he completed a II level master’s degree (MSc) in ‘Percutaneous interventional treatment of structural heart diseases’ at the Scuola Superiore Sant’Anna of Pisa. His areas of expertise include coronary, peripheral, and structural interventions. He is a faculty member at international scientific meetings (GISE, CCC, EuroPCR, C3, and CRT).

## Supplementary material

**Consent:** The authors confirm that the EHJ-CR Consent Form has been signed by the patient, and that the original signed form is held by the treating institution.

**Funding:** None declared.
